# Structural analysis of 2-iodo­benzamide and 2-iodo-*N*-phenyl­benzamide

**DOI:** 10.1107/S2056989018010162

**Published:** 2018-07-20

**Authors:** Keshab M. Bairagi, Vipin B. S. Kumar, Subhrajyoti Bhandary, Katharigatta N. Venugopala, Susanta K. Nayak

**Affiliations:** a Department of Chemistry, Visvesvaraya National Institute of Technology, Nagpur 440 010, Maharashtra, India; b Department of Chemistry, Indian Institute of Science Education and Research Bhopal, Bhauri, Bhopal 462 066, Madhya Pradesh, India; c Department of Biotechnology and Food Technology, Durban University of Technology, Durban 4001, South Africa

**Keywords:** crystal structure, benzamide, dimer, tetra­mer, hydrogen bonds, C—I⋯π(ring) inter­actions

## Abstract

The title compounds, 2-iodo­benzamide, C_7_H_6_INO (I), and 2-iodo-*N*-phenyl­benzamide, C_13_H_10_INO (II), were both synthesized from 2-iodo­benzoic acid. In the crystal structure of (I), N—H⋯O and hydrogen bonds form two sets of closed rings, generating dimers and tetra­mers. These combine with C—I⋯π(ring) halogen bonds to form sheets of mol­ecules in the *bc* plane. For (II), N—H⋯O hydrogen bonds form chains along the *a*-axis direction, while inversion-related C—I⋯π(ring) contacts supported by C—H⋯π(ring) interactions generate sheets of mol­ecules along the *ab* diagonal.

## Abstract 2

The mol­ecular and crystal structures of 2-iodo benzamide and 2-iodo-*N*-phenyl­benzamide are reported. In both crystals, N—H⋯O hydrogen bonds and C—I⋯π(ring) inter­actions stabilize the packing with additional C—H⋯π(ring) contacts found in the latter.

## Chemical context   

Aromatic amides can be found in a wide range of aromatic molecules and they also serve as inter­mediates in the production of many pharmaceutical compounds (Suchetan *et al.*, 2016 ▸). Aromatic amides and *N*-aryl amides display a wide spectrum of pharmacological properties and are used as anti­bacterial (Ragavan *et al.*, 2010 ▸), analgesic (Starmer *et al.*, 1971 ▸), anti­viral (Hu *et al.*, 2008 ▸), anti-inflammatory (Kalgutkar *et al.*, 2000 ▸) and anti-cancer (Pradidphol *et al.*, 2012 ▸) agents. Furthermore, *N*-aryl amides are known to act as anti-tumor agents against a broad spectrum of human tumors (Abdou *et al.*, 2004 ▸). In view of their potential importance, the title compounds (I) and (II) were synthesized and we report herein a comparison of their structures.
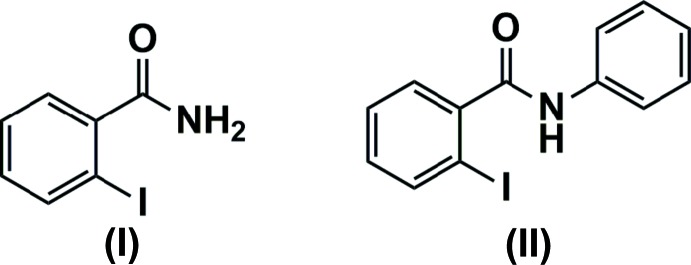


## Structural commentary   

**Figure 1:**
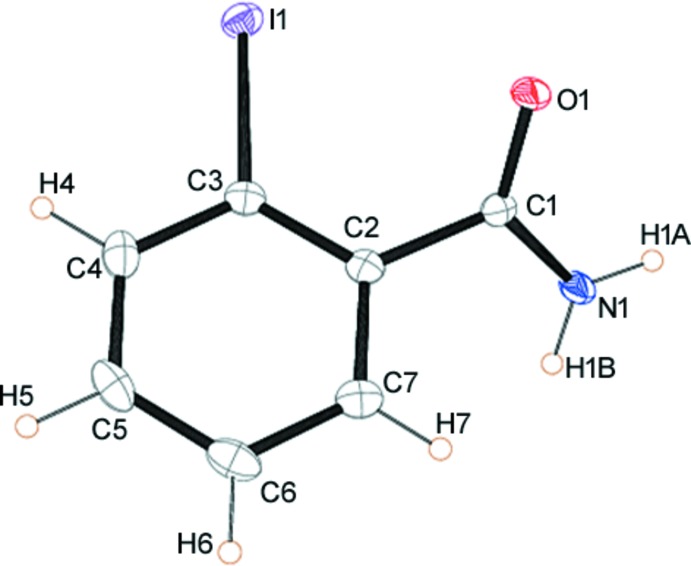
The mol­ecular structure of (I) showing the atom numbering with ellipsoids drawn at the 50% probability level.

Both compounds (I) and (II) crystallize with one mol­ecule in the asymmetric unit (*Z*′ = 1). The mol­ecular structures of the mol­ecules are shown in Figs. 1 ▸ and 2 ▸, respectively. In (I) the aromatic ring is inclined to the O1/C1/N1 plane of the amide by 44.37 (1)° whereas in (II) the two aromatic rings are almost orthogonal with an angle of 79.84 (6)° between them. The iodo­benzene ring plane is inclined to the O1/C1/N1 amide plane by 52.01 (1)°, somewhat similar to the inclination found for (I), while the phenyl ring of the amide is inclined by 28.45 (5)° to this plane.

## Supra­molecular features   

**Figure 3:**
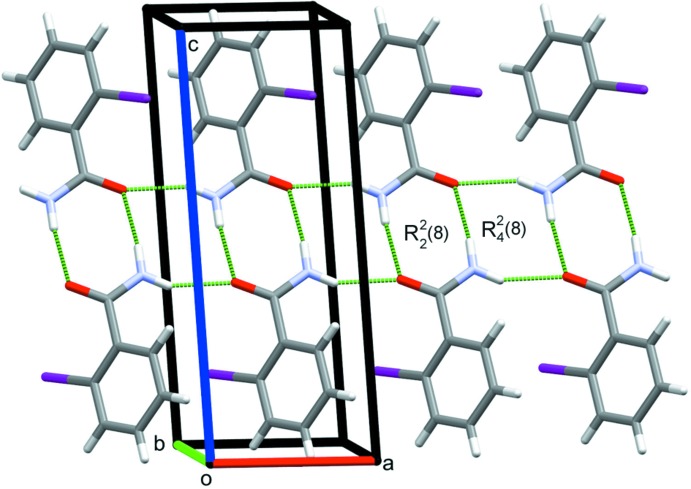
Chains of mol­ecules of (I) along the *a*-axis direction, showing the dimers and tetra­mers formed by N—H⋯O hydrogen bonds.

**Figure 4:**
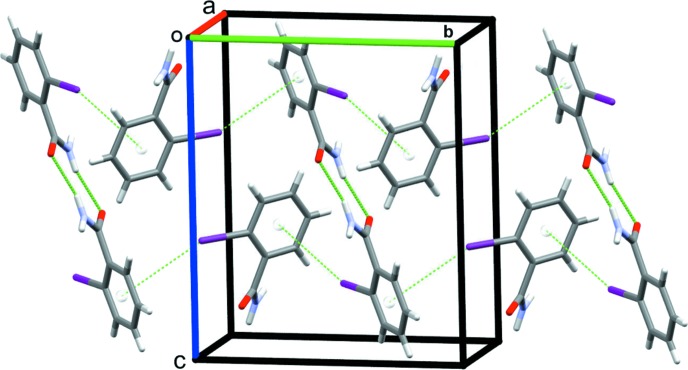
N—H⋯O and C—I⋯π(ring) contacts forming sheets of mol­ecules of (I) in the *bc* plane.

In the crystal structure of compound (I), strong classical N1—H1*A*⋯O1 and N1—H1*B*⋯O1 hydrogen bonds, Table 1 ▸, arrange the mol­ecules in two linked sets of closed rings, forming both dimers with an 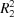
(8) graph-set motif and tetra­mers that enclose 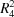
(8) rings (Etter *et al.*, 1990 ▸). These contacts form chains of mol­ecules along the *a*-axis direction (Fig. 3 ▸). In addition, C3—I1⋯*Cg*1 halogen bonds, Table 1 ▸, combine with the previously mentioned inversion dimers to generate sheets of mol­ecules in the *bc* plane (Fig. 4 ▸).

**Figure 5:**
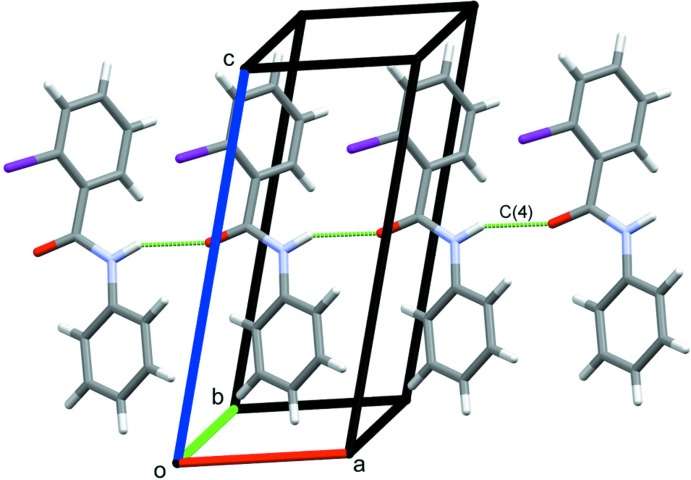
N—H⋯O hydrogen bonds forming chains of mol­ecules of (II) along the *a*-axis direction.

**Figure 6:**
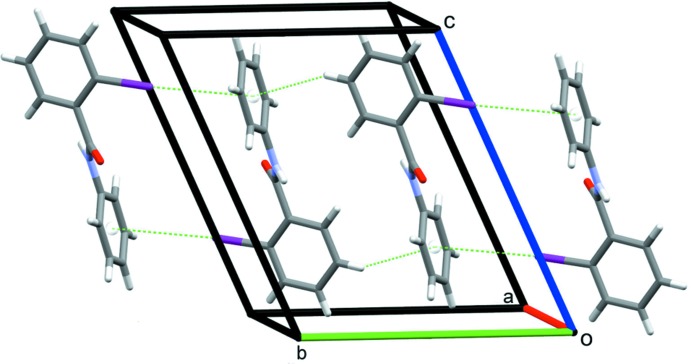
C—I⋯π(ring) and C—H⋯π(ring) contacts generating sheets of mol­ecules of (II) along the *ab* diagonal

For compound (II), the absence of a second H atom on the N1 amine nitro­gen atom limits the formation of classical hydrogen bonds to N1—H1⋯O1 contacts that generate *C*(4) mol­ecular chains along the *a*-axis direction (Fig. 5 ▸, Table 2 ▸). Additional weak inversion-related C3—I1⋯*Cg*2 inter­actions (Table 2 ▸), in this instance also supported by C6—H6⋯*Cg*2 contacts that also lie about an inversion centre, form sheets of mol­ecules along the *ab* diagonal (Fig. 6 ▸, Table 2 ▸).

## Database survey   

A search for the crystal structures of 2-iodo­benzamide and 2-iodo-*N*-phenyl­benzamide was carried out in the Cambridge Structural Database (Conquest Version 1.17; CSD Version 5.39, last update November 2017; Groom *et al.*, 2016 ▸). Compound (I) was found to have been previously reported from film data (IBNZAM; Nakata *et al.*, 1976 ▸), but there were no hits for compound (II). Four other related structures were observed: two fluorine-substituted 2-iodo­benzamides, FAHSAK and FAHSIS (Nayak *et al.*, 2012 ▸) and two nitro substituted 2-iodo­benzamides, TAQBIX (Garden *et al.*, 2005 ▸) and WAWMAJ (Wardell *et al.*, 2005 ▸).

## Synthesis and crystallization   

The synthesis of the title compounds was carried out using a reported procedure (Jursic & Zdravkovski, 1993 ▸; Kavala *et al.*, 2012 ▸; Mao *et al.*, 2012 ▸). Single crystals for both compounds were grown by the slow evaporation method from di­chloro­methane and hexane (*v*/*v* 1:1) at low temperature for (I), whereas those for compound (II) were obtained from aceto­nitrile solvent at room temperature. The melting points of (I) and (II) are 398.2 and 419.6 K, respectively. Infra-red (IR) spectra confirm the presence of various functional groups as follows: compound (I) (cm^−1^): N—H = 3362, 3177, C=O = 1644, C=C = 1581–1470, *ortho*-substituted ring = 734; compound (II) (cm^−1^): N—H = 3235, C*sp* ^2^—H = 3037, C=O = 1646, C=C = 1536–1488, *ortho*-substituted ring = 752, N—H bending = 1597.

## Refinement   

Crystal data, data collection and structure refinement details are summarized in Table 3 ▸. All H atoms were refined using a riding model with *d*(N—H) = 0.86 Å, *U*_iso_(H) = 1.2*U*_eq_(N) and *d*(C—H) = 0.93 Å, *U*_iso_(H) = 1.2*U*_eq_(C) for (I) and *d*(N—H) = 0.88 Å, *U*_iso_(H) = 1.2*U*_eq_(N) and *d*(C—H) = 0.95 Å, *U*_iso_(H) = 1.2*U*_eq_(C) for (II).

## Supplementary Material

Crystal structure: contains datablock(s) global, I, II. DOI: 10.1107/S2056989018010162/sj5558sup1.cif

Structure factors: contains datablock(s) I. DOI: 10.1107/S2056989018010162/sj5558Isup2.hkl

Structure factors: contains datablock(s) II. DOI: 10.1107/S2056989018010162/sj5558IIsup3.hkl

Supporting information file. DOI: 10.1107/S2056989018010162/sj5558Isup4.cml

Supporting information file. DOI: 10.1107/S2056989018010162/sj5558IIsup5.cml

CCDC references: 1855731, 1855730

Additional supporting information:  crystallographic information; 3D view; checkCIF report

## Figures and Tables

**Figure 2:**
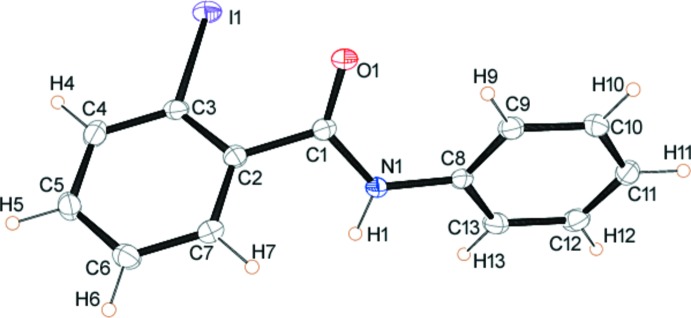
The mol­ecular structure of (II) showing the atom numbering with ellipsoids drawn at the 50% probability level.

**Table 1:** Hydrogen-bond geometry (Å, °) for (I) *Cg*1 is the centroid of the C2–C7 phenyl ring.

*D*—H⋯*A*	*D*—H	H⋯*A*	*D*⋯*A*	*D*—H⋯*A*
N1—H1*A*⋯O1^i^	0.86	2.11	2.951 (2)	164
N1—H1*B*⋯O1^ii^	0.86	2.05	2.843 (2)	154
C3—I1⋯*Cg*1^iii^	2.11 (1)	3.99 (1)	5.877 (2)	148 (1)

Symmetry codes: (i) 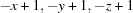; (ii) 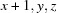; (iii) 
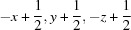
.

**Table 2:** Hydrogen-bond geometry (Å, °) for (II) *Cg*2 is the centroid of the C8–C13 benzene ring.

*D*—H⋯*A*	*D*—H	H⋯*A*	*D*⋯*A*	*D*—H⋯*A*
N1—H1⋯O1^i^	0.88	2.15	2.942 (2)	150
C3—I1⋯*Cg*2^ii^	2.10 (1)	3.83 (1)	5.816 (2)	156 (1)
C6—H6⋯*Cg*2^iii^	0.95	2.81	3.627 (2)	144

Symmetry codes: (i) 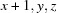; (ii) 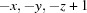; (iii) 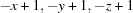.

**Table 3:** Experimental details

	(I)	(II)
Crystal data		
Chemical formula	C_7_H_6_INO	C_13_H_10_INO
*M*_r_	247.03	323.12
Crystal system, space group	Monoclinic, *P*2_1_/*n*	Triclinic, *P* 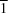
Temperature (K)	296	120
*a*, *b*, *c* (Å)	5.0531 (2), 11.4478 (5), 13.2945 (5)	5.1225 (2), 10.4572 (4), 12.2167 (5)
α, β, γ (°)	90, 93.245 (1), 90	66.034 (2), 78.882 (2), 85.760 (2)
*V* (Å^3^)	767.81 (5)	586.76 (4)
*Z*	4	2
Radiation type	Mo *K*α	Mo *K*α
μ (mm^−1^)	4.10	2.71
Crystal size (mm)	0.23 × 0.22 × 0.21	0.23 × 0.22 × 0.21

Data collection		
Diffractometer	Bruker Kappa APEXII DUO	Bruker Kappa APEXII DUO
Absorption correction	Multi-scan (*SADABS*; Bruker, 2014 ▸)	Multi-scan (*SADABS*; Bruker, 2014 ▸)
*T*_min_, *T*_max_	0.429, 0.456	0.546, 0.570
No. of measured, independent and observed [*I* > 2σ(*I*)] reflections	5827, 1504, 1461	13292, 2309, 2278
*R*_int_	0.021	0.018
(sin θ/λ)_max_ (Å^−1^)	0.617	0.617

Refinement		
*R*[*F* ^2^ > 2σ(*F* ^2^)], *wR*(*F* ^2^), *S*	0.014, 0.033, 1.16	0.017, 0.042, 1.08
No. of reflections	1504	2309
No. of parameters	92	145
H-atom treatment	H-atom parameters constrained	H-atom parameters constrained
Δρ_max_, Δρ_min_ (e Å^−3^)	0.45, −0.35	0.81, −0.48

Computer programs: *APEX2* and *SAINT* (Bruker, 2014 ▸), *SHELXS14* (Sheldrick, 2008 ▸), *SHELXL2014* (Sheldrick, 2015 ▸), *PLATON* (Spek, 2009 ▸), *Mercury* (Macrae *et al.*, 2008 ▸), *WinGX* (Farrugia, 2012 ▸) and *PARST* (Nardelli, 1995 ▸).

## supplementary crystallographic information

### 2-Iodobenzamide (I) Crystal data

**Table:**

C_7_H_6_INO	*F*(000) = 464
*M**_r_* = 247.03	*D*_x_ = 2.137 Mg m^−^^3^
Monoclinic, *P*2_1_/*n*	Mo *K*α radiation, λ = 0.71073 Å
*a* = 5.0531 (2) Å	Cell parameters from 1504 reflections
*b* = 11.4478 (5) Å	θ = 2.3–26.0°
*c* = 13.2945 (5) Å	µ = 4.10 mm^−^^1^
β = 93.245 (1)°	*T* = 296 K
*V* = 767.81 (5) Å^3^	Plate, colorless
*Z* = 4	0.23 × 0.22 × 0.21 mm

### 2-Iodobenzamide (I) Data collection

**Table:**

Bruker Kappa APEXII DUO diffractometer	1504 independent reflections
Radiation source: fine-focus sealed tube	1461 reflections with *I* > 2σ(*I*)
Graphite monochromator	*R*_int_ = 0.021
ω scans	θ_max_ = 26.0°, θ_min_ = 2.4°
Absorption correction: multi-scan (SADABS; Bruker, 2014)	*h* = −6→6
*T*_min_ = 0.429, *T*_max_ = 0.456	*k* = −14→11
5827 measured reflections	*l* = −15→16

### 2-Iodobenzamide (I) Refinement

**Table:**

Refinement on *F*^2^	Hydrogen site location: inferred from neighbouring sites
Least-squares matrix: full	H-atom parameters constrained
*R*[*F*^2^ > 2σ(*F*^2^)] = 0.014	*w* = 1/[σ^2^(*F*_o_^2^) + (0.0075*P*)^2^ + 0.6908*P*] where *P* = (*F*_o_^2^ + 2*F*_c_^2^)/3
*wR*(*F*^2^) = 0.033	(Δ/σ)_max_ = 0.002
*S* = 1.16	Δρ_max_ = 0.45 e Å^−^^3^
1504 reflections	Δρ_min_ = −0.35 e Å^−^^3^
92 parameters	Extinction correction: SHELXL2014 (Sheldrick, 2015), Fc^*^=kFc[1+0.001xFc^2^λ^3^/sin(2θ)]^-1/4^
0 restraints	Extinction coefficient: 0.0170 (5)

### 2-Iodobenzamide (I) Special details

**Table:**

Geometry. All esds (except the esd in the dihedral angle between two l.s. planes) are estimated using the full covariance matrix. The cell esds are taken into account individually in the estimation of esds in distances, angles and torsion angles; correlations between esds in cell parameters are only used when they are defined by crystal symmetry. An approximate (isotropic) treatment of cell esds is used for estimating esds involving l.s. planes.

### 2-Iodobenzamide (I) Fractional atomic coordinates and isotropic or equivalent isotropic displacement parameters (Å^2^)

**Table:**

	*x*	*y*	*z*	*U*_iso_*/*U*_eq_	
I1	0.14922 (2)	0.55570 (2)	0.18090 (2)	0.01703 (7)	
O1	0.3073 (3)	0.43218 (14)	0.39426 (11)	0.0177 (3)	
N1	0.7508 (3)	0.44020 (16)	0.41536 (14)	0.0168 (4)	
H1A	0.7438	0.4650	0.4762	0.020*	
H1B	0.9018	0.4297	0.3900	0.020*	
C5	0.6303 (4)	0.2793 (2)	0.06578 (17)	0.0219 (5)	
H5	0.6514	0.2473	0.0024	0.026*	
C6	0.7846 (4)	0.23997 (19)	0.14775 (17)	0.0202 (5)	
H6	0.9113	0.1824	0.1396	0.024*	
C7	0.7504 (4)	0.28652 (19)	0.24225 (17)	0.0165 (4)	
H7	0.8555	0.2598	0.2972	0.020*	
C2	0.5610 (4)	0.37276 (18)	0.25648 (15)	0.0125 (4)	
C1	0.5297 (4)	0.41830 (18)	0.36086 (15)	0.0125 (4)	
C4	0.4440 (4)	0.3662 (2)	0.07746 (16)	0.0193 (5)	
H4	0.3416	0.3931	0.0219	0.023*	
C3	0.4101 (4)	0.41317 (18)	0.17218 (16)	0.0138 (4)	

### 2-Iodobenzamide (I) Atomic displacement parameters (Å^2^)

**Table:**

	*U*^11^	*U*^22^	*U*^33^	*U*^12^	*U*^13^	*U*^23^
I1	0.01454 (9)	0.01736 (10)	0.01910 (10)	0.00237 (5)	0.00030 (5)	0.00353 (5)
O1	0.0082 (7)	0.0300 (9)	0.0152 (8)	−0.0007 (6)	0.0019 (5)	−0.0026 (6)
N1	0.0094 (8)	0.0280 (11)	0.0132 (9)	−0.0003 (7)	0.0021 (6)	−0.0044 (8)
C5	0.0286 (12)	0.0200 (11)	0.0177 (12)	−0.0038 (9)	0.0070 (9)	−0.0071 (9)
C6	0.0210 (11)	0.0125 (11)	0.0277 (12)	−0.0010 (9)	0.0085 (9)	−0.0048 (9)
C7	0.0138 (9)	0.0140 (10)	0.0220 (11)	−0.0022 (8)	0.0024 (8)	0.0013 (9)
C2	0.0100 (9)	0.0122 (10)	0.0154 (10)	−0.0034 (7)	0.0019 (7)	−0.0003 (8)
C1	0.0118 (9)	0.0117 (9)	0.0142 (10)	−0.0002 (8)	0.0015 (7)	0.0036 (8)
C4	0.0213 (10)	0.0226 (12)	0.0140 (11)	−0.0042 (9)	−0.0002 (8)	−0.0012 (9)
C3	0.0120 (9)	0.0122 (10)	0.0173 (11)	−0.0019 (8)	0.0025 (8)	0.0005 (8)

### 2-Iodobenzamide (I) Geometric parameters (Å, º)

**Table:**

I1—C3	2.105 (2)	C6—C7	1.385 (3)
O1—C1	1.242 (2)	C6—H6	0.9300
N1—C1	1.321 (3)	C7—C2	1.395 (3)
N1—H1A	0.8600	C7—H7	0.9300
N1—H1B	0.8600	C2—C3	1.398 (3)
C5—C6	1.379 (3)	C2—C1	1.499 (3)
C5—C4	1.384 (3)	C4—C3	1.389 (3)
C5—H5	0.9300	C4—H4	0.9300
			
C1—N1—H1A	120.0	C7—C2—C3	118.20 (19)
C1—N1—H1B	120.0	C7—C2—C1	118.73 (18)
H1A—N1—H1B	120.0	C3—C2—C1	123.07 (18)
C6—C5—C4	120.2 (2)	O1—C1—N1	122.29 (19)
C6—C5—H5	119.9	O1—C1—C2	121.37 (18)
C4—C5—H5	119.9	N1—C1—C2	116.32 (16)
C5—C6—C7	119.8 (2)	C5—C4—C3	120.0 (2)
C5—C6—H6	120.1	C5—C4—H4	120.0
C7—C6—H6	120.1	C3—C4—H4	120.0
C6—C7—C2	121.1 (2)	C4—C3—C2	120.61 (19)
C6—C7—H7	119.4	C4—C3—I1	117.38 (16)
C2—C7—H7	119.4	C2—C3—I1	121.81 (15)
			
C4—C5—C6—C7	0.9 (3)	C6—C5—C4—C3	−0.7 (3)
C5—C6—C7—C2	0.2 (3)	C5—C4—C3—C2	−0.6 (3)
C6—C7—C2—C3	−1.5 (3)	C5—C4—C3—I1	174.29 (16)
C6—C7—C2—C1	178.91 (18)	C7—C2—C3—C4	1.7 (3)
C7—C2—C1—O1	−135.1 (2)	C1—C2—C3—C4	−178.75 (18)
C3—C2—C1—O1	45.3 (3)	C7—C2—C3—I1	−172.99 (14)
C7—C2—C1—N1	43.5 (3)	C1—C2—C3—I1	6.6 (3)
C3—C2—C1—N1	−136.1 (2)		

### 2-Iodobenzamide (I) Hydrogen-bond geometry (Å, º)

Cg1 is the centroid of the C2–C7 phenyl ring.

**Table:**

*D*—H···*A*	*D*—H	H···*A*	*D*···*A*	*D*—H···*A*
N1—H1*A*···O1^i^	0.86	2.11	2.951 (2)	164
N1—H1*B*···O1^ii^	0.86	2.05	2.843 (2)	154
C3—I1···*Cg*1^iii^	2.11 (1)	3.99 (1)	5.877 (2)	148 (1)

Symmetry codes: (i) −*x*+1, −*y*+1, −*z*+1; (ii) *x*+1, *y*, *z*; (iii) −*x*+1/2, *y*+1/2, −*z*+1/2.

### 2-Iodo-N-phenylbenzamide (II) Crystal data

**Table:**

C_13_H_10_INO	*Z* = 2
*M**_r_* = 323.12	*F*(000) = 312
Triclinic, *P*1	*D*_x_ = 1.829 Mg m^−^^3^
*a* = 5.1225 (2) Å	Mo *K*α radiation, λ = 0.71073 Å
*b* = 10.4572 (4) Å	Cell parameters from 2309 reflections
*c* = 12.2167 (5) Å	θ = 1.9–26.0°
α = 66.034 (2)°	µ = 2.71 mm^−^^1^
β = 78.882 (2)°	*T* = 120 K
γ = 85.760 (2)°	Plate, colorless
*V* = 586.76 (4) Å^3^	0.23 × 0.22 × 0.21 mm

### 2-Iodo-N-phenylbenzamide (II) Data collection

**Table:**

Bruker Kappa APEXII DUO diffractometer	2309 independent reflections
Radiation source: fine-focus sealed tube	2278 reflections with *I* > 2σ(*I*)
Graphite monochromator	*R*_int_ = 0.018
ω scans	θ_max_ = 26.0°, θ_min_ = 1.9°
Absorption correction: multi-scan (SADABS; Bruker, 2014)	*h* = −6→6
*T*_min_ = 0.546, *T*_max_ = 0.570	*k* = −12→12
13292 measured reflections	*l* = −15→14

### 2-Iodo-N-phenylbenzamide (II) Refinement

**Table:**

Refinement on *F*^2^	0 restraints
Least-squares matrix: full	Hydrogen site location: inferred from neighbouring sites
*R*[*F*^2^ > 2σ(*F*^2^)] = 0.017	H-atom parameters constrained
*wR*(*F*^2^) = 0.042	*w* = 1/[σ^2^(*F*_o_^2^) + (0.0207*P*)^2^ + 0.7193*P*] where *P* = (*F*_o_^2^ + 2*F*_c_^2^)/3
*S* = 1.08	(Δ/σ)_max_ < 0.001
2309 reflections	Δρ_max_ = 0.81 e Å^−^^3^
145 parameters	Δρ_min_ = −0.48 e Å^−^^3^

### 2-Iodo-N-phenylbenzamide (II) Special details

**Table:**

Geometry. All esds (except the esd in the dihedral angle between two l.s. planes) are estimated using the full covariance matrix. The cell esds are taken into account individually in the estimation of esds in distances, angles and torsion angles; correlations between esds in cell parameters are only used when they are defined by crystal symmetry. An approximate (isotropic) treatment of cell esds is used for estimating esds involving l.s. planes.

### 2-Iodo-N-phenylbenzamide (II) Fractional atomic coordinates and isotropic or equivalent isotropic displacement parameters (Å^2^)

**Table:**

	*x*	*y*	*z*	*U*_iso_*/*U*_eq_	
I1	−0.30400 (3)	0.03723 (2)	0.77480 (2)	0.01972 (6)	
O1	−0.1224 (3)	0.31161 (18)	0.51921 (14)	0.0233 (3)	
N1	0.3285 (3)	0.2852 (2)	0.49180 (16)	0.0169 (4)	
H1	0.4668	0.2731	0.5279	0.020*	
C1	0.0875 (4)	0.2939 (2)	0.55727 (19)	0.0161 (4)	
C2	0.0968 (4)	0.2802 (2)	0.68392 (19)	0.0148 (4)	
C3	−0.0677 (4)	0.1861 (2)	0.7861 (2)	0.0156 (4)	
C4	−0.0629 (4)	0.1793 (2)	0.9014 (2)	0.0194 (4)	
H4	−0.1751	0.1151	0.9704	0.023*	
C5	0.1069 (4)	0.2670 (2)	0.9157 (2)	0.0206 (4)	
H5	0.1091	0.2632	0.9945	0.025*	
C6	0.2728 (4)	0.3597 (2)	0.8154 (2)	0.0198 (4)	
H6	0.3893	0.4191	0.8255	0.024*	
C7	0.2685 (4)	0.3657 (2)	0.7005 (2)	0.0169 (4)	
H7	0.3838	0.4289	0.6321	0.020*	
C8	0.3800 (4)	0.2938 (2)	0.37055 (19)	0.0159 (4)	
C9	0.2215 (4)	0.3717 (2)	0.2855 (2)	0.0180 (4)	
H9	0.0683	0.4185	0.3083	0.022*	
C10	0.2897 (4)	0.3802 (2)	0.1671 (2)	0.0191 (4)	
H10	0.1821	0.4334	0.1089	0.023*	
C11	0.5124 (4)	0.3123 (2)	0.1323 (2)	0.0203 (4)	
H11	0.5579	0.3190	0.0510	0.024*	
C12	0.6677 (4)	0.2343 (2)	0.2180 (2)	0.0209 (5)	
H12	0.8204	0.1873	0.1952	0.025*	
C13	0.6024 (4)	0.2245 (2)	0.3364 (2)	0.0192 (4)	
H13	0.7094	0.1703	0.3945	0.023*	

### 2-Iodo-N-phenylbenzamide (II) Atomic displacement parameters (Å^2^)

**Table:**

	*U*^11^	*U*^22^	*U*^33^	*U*^12^	*U*^13^	*U*^23^
I1	0.01580 (8)	0.01876 (9)	0.02535 (9)	−0.00291 (5)	−0.00378 (6)	−0.00904 (6)
O1	0.0114 (7)	0.0402 (10)	0.0194 (8)	−0.0006 (7)	−0.0035 (6)	−0.0126 (7)
N1	0.0106 (8)	0.0271 (10)	0.0150 (9)	0.0003 (7)	−0.0026 (7)	−0.0103 (8)
C1	0.0134 (10)	0.0183 (10)	0.0171 (10)	−0.0018 (8)	−0.0013 (8)	−0.0078 (8)
C2	0.0118 (9)	0.0171 (10)	0.0169 (10)	0.0039 (8)	−0.0035 (8)	−0.0084 (8)
C3	0.0109 (9)	0.0172 (10)	0.0214 (11)	0.0004 (8)	−0.0028 (8)	−0.0105 (9)
C4	0.0178 (10)	0.0221 (11)	0.0160 (10)	0.0002 (8)	0.0004 (8)	−0.0069 (9)
C5	0.0207 (11)	0.0270 (12)	0.0174 (10)	0.0026 (9)	−0.0042 (8)	−0.0124 (9)
C6	0.0186 (10)	0.0216 (11)	0.0235 (11)	0.0002 (8)	−0.0063 (9)	−0.0124 (9)
C7	0.0131 (10)	0.0180 (10)	0.0187 (10)	−0.0005 (8)	−0.0013 (8)	−0.0070 (9)
C8	0.0129 (9)	0.0206 (10)	0.0161 (10)	−0.0047 (8)	−0.0003 (8)	−0.0095 (9)
C9	0.0139 (10)	0.0213 (11)	0.0203 (11)	−0.0010 (8)	−0.0018 (8)	−0.0103 (9)
C10	0.0179 (10)	0.0220 (11)	0.0179 (10)	−0.0045 (8)	−0.0046 (8)	−0.0071 (9)
C11	0.0208 (11)	0.0247 (11)	0.0182 (10)	−0.0076 (9)	0.0010 (8)	−0.0121 (9)
C12	0.0152 (10)	0.0256 (12)	0.0259 (12)	−0.0031 (9)	0.0014 (9)	−0.0159 (10)
C13	0.0138 (10)	0.0244 (11)	0.0218 (11)	0.0004 (8)	−0.0046 (8)	−0.0109 (9)

### 2-Iodo-N-phenylbenzamide (II) Geometric parameters (Å, º)

**Table:**

I1—C3	2.104 (2)	C6—H6	0.9500
O1—C1	1.225 (3)	C7—H7	0.9500
N1—C1	1.354 (3)	C8—C13	1.392 (3)
N1—C8	1.420 (3)	C8—C9	1.394 (3)
N1—H1	0.8800	C9—C10	1.388 (3)
C1—C2	1.505 (3)	C9—H9	0.9500
C2—C7	1.395 (3)	C10—C11	1.388 (3)
C2—C3	1.399 (3)	C10—H10	0.9500
C3—C4	1.387 (3)	C11—C12	1.388 (3)
C4—C5	1.390 (3)	C11—H11	0.9500
C4—H4	0.9500	C12—C13	1.382 (3)
C5—C6	1.385 (3)	C12—H12	0.9500
C5—H5	0.9500	C13—H13	0.9500
C6—C7	1.384 (3)		
			
C1—N1—C8	126.37 (18)	C6—C7—C2	120.8 (2)
C1—N1—H1	116.8	C6—C7—H7	119.6
C8—N1—H1	116.8	C2—C7—H7	119.6
O1—C1—N1	124.4 (2)	C13—C8—C9	119.80 (19)
O1—C1—C2	121.64 (19)	C13—C8—N1	117.79 (19)
N1—C1—C2	113.98 (18)	C9—C8—N1	122.38 (19)
C7—C2—C3	118.70 (19)	C10—C9—C8	119.4 (2)
C7—C2—C1	119.60 (19)	C10—C9—H9	120.3
C3—C2—C1	121.68 (18)	C8—C9—H9	120.3
C4—C3—C2	120.61 (19)	C11—C10—C9	121.1 (2)
C4—C3—I1	117.07 (16)	C11—C10—H10	119.5
C2—C3—I1	122.08 (15)	C9—C10—H10	119.5
C3—C4—C5	119.7 (2)	C10—C11—C12	119.0 (2)
C3—C4—H4	120.1	C10—C11—H11	120.5
C5—C4—H4	120.1	C12—C11—H11	120.5
C6—C5—C4	120.3 (2)	C13—C12—C11	120.7 (2)
C6—C5—H5	119.9	C13—C12—H12	119.7
C4—C5—H5	119.9	C11—C12—H12	119.7
C7—C6—C5	119.9 (2)	C12—C13—C8	120.1 (2)
C7—C6—H6	120.1	C12—C13—H13	120.0
C5—C6—H6	120.1	C8—C13—H13	120.0
			
C8—N1—C1—O1	−0.6 (4)	C5—C6—C7—C2	0.6 (3)
C8—N1—C1—C2	179.69 (19)	C3—C2—C7—C6	−1.2 (3)
O1—C1—C2—C7	−127.2 (2)	C1—C2—C7—C6	177.16 (19)
N1—C1—C2—C7	52.6 (3)	C1—N1—C8—C13	−152.1 (2)
O1—C1—C2—C3	51.1 (3)	C1—N1—C8—C9	29.9 (3)
N1—C1—C2—C3	−129.1 (2)	C13—C8—C9—C10	−0.7 (3)
C7—C2—C3—C4	0.9 (3)	N1—C8—C9—C10	177.34 (19)
C1—C2—C3—C4	−177.47 (19)	C8—C9—C10—C11	0.1 (3)
C7—C2—C3—I1	−173.28 (15)	C9—C10—C11—C12	0.3 (3)
C1—C2—C3—I1	8.4 (3)	C10—C11—C12—C13	−0.1 (3)
C2—C3—C4—C5	0.1 (3)	C11—C12—C13—C8	−0.4 (3)
I1—C3—C4—C5	174.51 (16)	C9—C8—C13—C12	0.8 (3)
C3—C4—C5—C6	−0.7 (3)	N1—C8—C13—C12	−177.3 (2)
C4—C5—C6—C7	0.4 (3)		

### 2-Iodo-N-phenylbenzamide (II) Hydrogen-bond geometry (Å, º)

Cg2 is the centroid of the C8–C13 benzene ring.

**Table:**

*D*—H···*A*	*D*—H	H···*A*	*D*···*A*	*D*—H···*A*
N1—H1···O1^i^	0.88	2.15	2.942 (2)	150
C3—I1···*Cg*2^ii^	2.10 (1)	3.83 (1)	5.816 (2)	156 (1)
C6—H6···*Cg*2^iii^	0.95	2.81	3.627 (2)	144

Symmetry codes: (i) *x*+1, *y*, *z*; (ii) −*x*, −*y*, −*z*+1; (iii) −*x*+1, −*y*+1, −*z*+1.
